# Supplementary material for: Determination of the Popularity of Dietary Supplements Using Google Search Rankings
Source: Nutrients. 2020 Mar 26;12(4):908. doi: 10.3390/nu12040908 (PMC7231191; doi:10.3390/nu12040908)
Supplement: Supplementary file 1 [file nutrients-12-00908-s001.pdf]

Supplementary file

Table S1

a) Checklist for Documentation of Google Trends research.

| Section/Topic | Checklist item |
|---|---|
| **Search Variables** | |
| Access Date | 17 December 2019 |
| Time Period | From January 2004 to 30 November 2019 |
| Query Category | Health |
| Region | Worldwide |
| Countries with Low Search Volume | Excluded |
| **Search Input** | |
| Non-adjusted | All n = 200 topics. The list is presented in section b). |
| Adjusted | Topics: "Lutein" +  separately all remain topics |
| **Rationale for Search Strategy** | |
| For Search Input | The searched topics are related to essential ingredients of dietary supplements. Because Google Trends enables to compare only five inputs at once we compared relative search volume of all topics with topic „Lutein" (adjusted data). Therefore, we were able to compare all topics in further analysis. |
| For Setting Chosen | We chose the only „Health" category to obtain only searches related to health informations. This was crucial to diminish significance of topics that may be associated with industry e.g. „Potassium". We excluded countries with low search volume which may be sensitive for outliers |

b) The list of n = 200 topics related to dietary supplements and their categories. Alphabetical order.

| No | Topic | Category |
|---|---|---|
| 1 | 4-Aminobenzoic acid | Chemical |
| 2 | 5-Hydroxytryptophan | Chemical |
| 3 | Acai berry | Botanical |
| 4 | Acetylcarnitine | Chemical |
| 5 | Acetylcysteine | Amino Acid |
| 6 | Alanine | Amino Acid |
| 7 | Alfalfa | Botanical |
| 8 | Aloe vera | Botanical |
| 9 | Alpha-galactosidase | Enzyme |
| 10 | alpha-Linolenic acid | Fat and fatty acid |
| 11 | Amylase | Enzyme |
| 12 | Arginine | Amino Acid |
| 13 | Artichoke | Botanical |
| 14 | Ashwagandha | Botanical |
| 15 | Astaxanthin | Chemical |
| 16 | Barbados Cherry | Botanical |
| 17 | Bearberry | Botanical |
| 18 | Beetroot | Botanical |
| 19 | Bell pepper | Botanical |
| 20 | beta-Carotene | Botanical |
| 21 | Betaine | Chemical |
| 22 | Bilberry | Botanical |
| 23 | Biotin | Vitamin |
| 24 | Bitter orange | Botanical |
| 25 | Black cohosh | Botanical |
| 26 | Black pepper | Botanical |
| 27 | Blackberry | Botanical |

| | | |
|---|---|---|
| 28 | Blackcurrant | Botanical |
| 29 | Blueberry | Botanical |
| 30 | Boron | Mineral |
| 31 | Branched-chain amino acid | Amino Acid |
| 32 | Broccoli | Botanical |
| 33 | Bromelain | Enzyme |
| 34 | Brussels sprout | Botanical |
| 35 | Burdock | Botanical |
| 36 | Caffeine | Chemical |
| 37 | Calcium | Mineral |
| 38 | Carnitine | Amino Acid |
| 39 | Cayenne pepper | Botanical |
| 40 | Centella asiatica | Botanical |
| 41 | Chlorella | Botanical |
| 42 | Chlorophyll | Botanical |
| 43 | Chromium | Mineral |
| 44 | Choline | Vitamin |
| 45 | Chondroitin sulfate | Chemical |
| 46 | Cinnamon | Botanical |
| 47 | Citrulline | Amino Acid |
| 48 | Cobalamin | Vitamin |
| 49 | Common Nettle | Botanical |
| 50 | Coneflower | Botanical |
| 51 | Conjugated linoleic acid | Fat and fatty acid |
| 52 | Copper | Mineral |
| 53 | Cordyceps | Botanical |
| 54 | Cranberry | Botanical |
| 55 | Creatine | Chemical |
| 56 | Curly kale | Botanical |

| | | |
|---|---|---|
| 57 | Cysteine | Amino Acid |
| 58 | Dandelion | Botanical |
| 59 | Dietary fiber | Fiber |
| 60 | Dimethylethanolamine | Chemical |
| 61 | Dipeptidyl peptidase-4 | Enzyme |
| 62 | Docosahexaenoic acid | Fat and fatty acid |
| 63 | Eicosapentaenoic acid | Fat and fatty acid |
| 64 | Female ginseng | Botanical |
| 65 | Fennel | Botanical |
| 66 | Fenugreek | Botanical |
| 67 | Field horsetail | Botanical |
| 68 | Fish oil | Fat and fatty acid |
| 69 | Five-flavor berry | Botanical |
| 70 | Flavonoid | Chemical |
| 71 | Flax seed | Botanical |
| 72 | Folate | Vitamin |
| 73 | Fructooligosaccharide | Botanical |
| 74 | gamma-Aminobutyric acid | Chemical |
| 75 | gamma-Linolenic acid | Fat and fatty acid |
| 76 | Garcinia cambogia | Botanical |
| 77 | Garlic | Botanical |
| 78 | Ginger | Botanical |
| 79 | Glucosamine | Chemical |
| 80 | Glutamic acid | Amino Acid |
| 81 | Glutamine | Amino Acid |
| 82 | Glutathione | Chemical |
| 83 | Glycine | Amino Acid |
| 84 | Goji | Botanical |
| 85 | Goldenseal | Botanical |

| | | |
|---|---|---|
| 86 | Grape seed extract | Botanical |
| 87 | Green coffee | Botanical |
| 88 | Green tea | Botanical |
| 89 | Guarana | Botanical |
| 90 | Hawthorn | Botanical |
| 91 | Hen-of-the-wood | Botanical |
| 92 | Hesperidin | Botanical |
| 93 | Histidine | Amino Acid |
| 94 | Holy Basil | Botanical |
| 95 | Hops | Botanical |
| 96 | Hyaluronic acid | Chemical |
| 97 | Indian frankincense | Botanical |
| 98 | Indian gooseberry | Botanical |
| 99 | Inositol | Vitamin |
| 100 | Iodine | Mineral |
| 101 | Iron | Mineral |
| 102 | Isoleucine | Amino Acid |
| 103 | Kelp | Botanical |
| 104 | Lactase | Enzyme |
| 105 | Lecithin | Fat and fatty acid |
| 106 | Lemon balm | Botanical |
| 107 | Leucine | Amino Acid |
| 108 | Lingzhi mushroom | Botanical |
| 109 | Linoleic acid | Fat and fatty acid |
| 110 | Linseed oil | Fat and fatty acid |
| 111 | Lipase | Enzyme |
| 112 | Lipoic acid | Chemical |
| 113 | Liquorice | Botanical |
| 114 | Lutein | Vitamin |

| | | |
|---|---|---|
| 115 | Lycopene | Botanical |
| 116 | Lysine | Amino Acid |
| 117 | Maca | Botanical |
| 118 | Magnesium | Mineral |
| 119 | Maidenhair tree | Botanical |
| 120 | Manganese | Mineral |
| 121 | Mangosteen | Botanical |
| 122 | Marsh mallow | Botanical |
| 123 | Melatonin | Chemical |
| 124 | Methionine | Amino Acid |
| 125 | Methylsulfonylmethane | Chemical |
| 126 | Milk thistle | Botanical |
| 127 | Milkvetch | Botanical |
| 128 | Molybdenum | Mineral |
| 129 | Monounsaturated fat | Fat and fatty acid |
| 130 | Niacin | Vitamin |
| 131 | Nickel | Mineral |
| 132 | Oat | Botanical |
| 133 | Oleic acid | Fat and fatty acid |
| 134 | Olive | Botanical |
| 135 | Omega-3 fatty acids | Fat and fatty acid |
| 136 | Omega-6 fatty acid | Fat and fatty acid |
| 137 | Omega-9 fatty acid | Fat and fatty acid |
| 138 | Oregano | Botanical |
| 139 | Pantothenic acid | Vitamin |
| 140 | Papain | Botanical |
| 141 | Papaya | Botanical |
| 142 | Parsley | Botanical |
| 143 | Passion fruit | Botanical |

| | | |
|---|---|---|
| 144 | Pectin | Fiber |
| 145 | Peppermint | Botanical |
| 146 | Phenylalanine | Amino Acid |
| 147 | Phosphatidylcholine | Fat and fatty acid |
| 148 | Phosphatidylserine | Fat and fatty acid |
| 149 | Phosphorus | Mineral |
| 150 | Polyunsaturated fat | Fat and fatty acid |
| 151 | Pomegranate | Botanical |
| 152 | Potassium | Mineral |
| 153 | Prebiotic | Bacterial |
| 154 | Probiotic | Bacterial |
| 155 | Proline | Amino Acid |
| 156 | Protease | Enzyme |
| 157 | Protein | Protein |
| 158 | Quercetin | Botanical |
| 159 | Raspberry | Botanical |
| 160 | Red Clover | Botanical |
| 161 | Red raspberry | Botanical |
| 162 | Resveratrol | Chemical |
| 163 | Rhodiola rosea | Botanical |
| 164 | Riboflavin | Vitamin |
| 165 | Rose hip | Botanical |
| 166 | Rosemary | Botanical |
| 167 | Rutin | Botanical |
| 168 | Saturated fat | Fat and fatty acid |
| 169 | Saw palmetto extract | Botanical |
| 170 | Selenium | Mineral |
| 171 | Siberian Ginseng | Botanical |
| 172 | Silicon | Mineral |

| | | |
|---|---|---|
| 173 | Slippery elm | Botanical |
| 174 | Spirulina | Botanical |
| 175 | ß-Alanine | Amino Acid |
| 176 | Stevia | Botanical |
| 177 | Sucrase | Enzyme |
| 178 | Synbiotics | Bacterial |
| 179 | Taurine | Amino Acid |
| 180 | Theanine | Amino Acid |
| 181 | Thiamine | Vitamin |
| 182 | Threonine | Amino Acid |
| 183 | Tryptophan | Amino Acid |
| 184 | Turmeric | Botanical |
| 185 | Tyrosine | Amino Acid |
| 186 | Valerian | Botanical |
| 187 | Valine | Amino Acid |
| 188 | Vanadium | Mineral |
| 189 | Velvet bean | Botanical |
| 190 | Vitamin A | Vitamin |
| 191 | Vitamin B-6 | Vitamin |
| 192 | Vitamin C | Vitamin |
| 193 | Vitamin D | Vitamin |
| 194 | Vitamin E | Vitamin |
| 195 | Vitamin K | Vitamin |
| 196 | Vitamin K2 | Vitamin |
| 197 | Whey protein | Protein |
| 198 | Yohimbe | Botanical |
| 199 | Zeaxanthin | Chemical |
| 200 | Zinc | Mineral |

Table S2
Use of data generated by Google Trends.

| | Interest over time | Region |
|---|---|---|
| | | Interest by region |
| Non-adjusted data | Time series analysis: yearly trends, seasonal variation (Table 3, Figures 2-3; Supplementary Table 5) | - |
| | | Compared breakdown by region |
| Adjusted data | Proportion of mean RSV to topic „Lutein” (Table 1, 2; Supplementary Table 3) | List of the most common topics in a specific country (Figure 1, Supplementary Table 4) |

RSV – Relative Search Volume

Table S3
Popularity of of dietary supplement-related topics in proprtion to “Lutein” (adjusted data; Relative Search Volume [RSV] over time).

| No | Topic | Proportion of RSV to Lutein |
|---|---|---|
| 1 | Magnesium | 23.72 |
| 2 | Protein | 15.22 |
| 3 | Iron | 15.12 |
| 4 | Calcium | 13.74 |
| 5 | Vitamin D | 12.22 |
| 6 | Potassium | 9.71 |
| 7 | Cobalamin | 9.36 |
| 8 | Caffeine | 8.06 |
| 9 | Vitamin C | 7.38 |
| 10 | Omega-3 fatty acids | 6.80 |
| 11 | Creatine | 6.77 |
| 12 | Folate | 6.67 |
| 13 | Zinc | 6.36 |
| 14 | Aloe vera | 6.11 |
| 15 | Ginger | 5.91 |
| 16 | Dietary fiber | 5.67 |
| 17 | Garlic | 5.63 |
| 18 | Iodine | 4.89 |
| 19 | Whey protein | 4.05 |
| 20 | Vitamin E | 3.86 |
| 21 | Fish oil | 3.79 |
| 22 | Turmeric | 3.71 |
| 23 | Probiotic | 3.70 |
| 24 | Vitamin A | 3.36 |
| 25 | Glucosamine | 3.33 |
| 26 | Maidenhair tree | 3.26 |
| 27 | Green tea | 3.23 |
| 28 | Carnitine | 3.18 |
| 29 | Copper | 3.15 |
| 30 | Maca | 2.85 |
| 31 | Vitamin B-6 | 2.80 |
| 32 | Vitamin K | 2.78 |
| 33 | Methylsulfonylmethane | 2.76 |
| 34 | Melatonin | 2.70 |
| 35 | Olive | 2.60 |
| 36 | Branched-chain amino acid | 2.56 |
| 37 | Niacin | 2.55 |
| 38 | Arginine | 2.52 |
| 39 | Cinnamon | 2.31 |
| 40 | Spirulina | 2.17 |
| 41 | Oat | 2.03 |
| 42 | Acetylcysteine | 1.98 |
| 43 | Valerian | 1.97 |
| 44 | Biotin | 1.89 |
| 45 | Thiamine | 1.71 |
| 46 | Glutathione | 1.70 |
| 47 | Fenugreek | 1.69 |

| | | |
|---|---|---|
| 48 | Milk thistle | 1.66 |
| 49 | Glutamine | 1.63 |
| 50 | Phosphorus | 1.59 |
| 51 | Conjugated linoleic acid | 1.56 |
| 52 | Hyaluronic acid | 1.51 |
| 53 | Lecithin | 1.48 |
| 54 | Linseed oil | 1.47 |
| 55 | Coneflower | 1.43 |
| 56 | Selenium | 1.39 |
| 57 | Lysine | 1.37 |
| 58 | Papaya | 1.35 |
| 59 | Flax seed | 1.34 |
| 60 | Saturated fat | 1.33 |
| 61 | Parsley | 1.28 |
| 62 | Fennel | 1.27 |
| 63 | Cranberry | 1.26 |
| 64 | Chromium | 1.24 |
| 65 | Rosemary | 1.23 |
| 66 | 5-Hydroxytryptophan | 1.23 |
| 67 | Liquorice | 1.22 |
| 68 | Pomegranate | 1.22 |
| 69 | Blueberry | 1.18 |
| 70 | Tryptophan | 1.18 |
| 71 | gamma-Aminobutyric acid | 1.13 |
| 72 | Amylase | 1.12 |
| 73 | Beetroot | 1.12 |
| 74 | Common Nettle | 1.09 |
| 75 | Raspberry | 1.08 |
| 76 | Riboflavin | 1.05 |
| 77 | Oregano | 1.01 |
| 78 | Silicon | 1.01 |
| 79 | Lutein | 1.00 |
| 80 | Lemon balm | 0.96 |
| 81 | Phenylalanine | 0.95 |
| 82 | Artichoke | 0.95 |
| 83 | Lipoic acid | 0.93 |
| 84 | Lipase | 0.90 |
| 85 | Garcinia cambogia | 0.87 |
| 86 | Goji | 0.86 |
| 87 | Peppermint | 0.84 |
| 88 | Chondroitin sulfate | 0.82 |
| 89 | Lingzhi mushroom | 0.81 |
| 90 | Mangosteen | 0.81 |
| 91 | Docosahexaenoic acid | 0.80 |
| 92 | Tyrosine | 0.79 |
| 93 | Rhodiola rosea | 0.79 |
| 94 | Dandelion | 0.77 |
| 95 | Protease | 0.74 |
| 96 | Choline | 0.73 |
| 97 | Flavonoid | 0.73 |
| 98 | Taurine | 0.73 |
| 99 | Ashwagandha | 0.70 |

| | | |
|---|---|---|
| 100 | Hawthorn | 0.69 |
| 101 | Nickel | 0.69 |
| 102 | Cayenne pepper | 0.68 |
| 103 | Black pepper | 0.66 |
| 104 | Pantothenic acid | 0.65 |
| 105 | Inositol | 0.65 |
| 106 | Saw palmetto extract | 0.65 |
| 107 | Bell pepper | 0.61 |
| 108 | Centella asiatica | 0.58 |
| 109 | Stevia | 0.55 |
| 110 | Black cohosh | 0.54 |
| 111 | Omega-6 fatty acid | 0.54 |
| 112 | Chlorophyll | 0.54 |
| 113 | Passion fruit | 0.54 |
| 114 | beta-Carotene | 0.52 |
| 115 | Glycine | 0.50 |
| 116 | Manganese | 0.48 |
| 117 | Bromelain | 0.48 |
| 118 | Chlorella | 0.47 |
| 119 | Rose hip | 0.45 |
| 120 | Curly kale | 0.45 |
| 121 | Lactase | 0.45 |
| 122 | Field horsetail | 0.43 |
| 123 | Cysteine | 0.42 |
| 124 | Theanine | 0.41 |
| 125 | Guarana | 0.41 |
| 126 | Female ginseng | 0.40 |
| 127 | Milkvetch | 0.40 |
| 128 | Lycopene | 0.39 |
| 129 | Glutamic acid | 0.39 |
| 130 | Eicosapentaenoic acid | 0.38 |
| 131 | Resveratrol | 0.38 |
| 132 | Cordyceps | 0.38 |
| 133 | Boron | 0.38 |
| 134 | Grape seed extract | 0.37 |
| 135 | Linoleic acid | 0.37 |
| 136 | Quercetin | 0.37 |
| 137 | Astaxanthin | 0.37 |
| 138 | Phosphatidylcholine | 0.37 |
| 139 | Goldenseal | 0.35 |
| 140 | Alfalfa | 0.34 |
| 141 | Pectin | 0.34 |
| 142 | Acai berry | 0.33 |
| 143 | Citrulline | 0.32 |
| 144 | Acetylcarnitine | 0.32 |
| 145 | Burdock | 0.32 |
| 146 | Methionine | 0.32 |
| 147 | Polyunsaturated fat | 0.31 |
| 148 | Kelp | 0.31 |
| 149 | Blackberry | 0.30 |
| 150 | Holy Basil | 0.30 |
| 151 | Prebiotic | 0.30 |

| | | |
|---|---|---|
| 152 | Siberian Ginseng | 0.30 |
| 153 | Monounsaturated fat | 0.29 |
| 154 | Phosphatidylserine | 0.29 |
| 155 | Bilberry | 0.28 |
| 156 | Leucine | 0.28 |
| 157 | Vitamin K2 | 0.27 |
| 158 | Oleic acid | 0.27 |
| 159 | Five-flavor berry | 0.27 |
| 160 | Red Clover | 0.26 |
| 161 | ß-Alanine | 0.26 |
| 162 | Blackcurrant | 0.26 |
| 163 | Red raspberry | 0.25 |
| 164 | Betaine | 0.24 |
| 165 | Indian gooseberry | 0.22 |
| 166 | Rutin | 0.21 |
| 167 | 4-Aminobenzoic acid | 0.21 |
| 168 | Molybdenum | 0.21 |
| 169 | Velvet bean | 0.21 |
| 170 | Broccoli | 0.20 |
| 171 | Papain | 0.19 |
| 172 | Slippery elm | 0.19 |
| 173 | Alanine | 0.18 |
| 174 | alpha-Linolenic acid | 0.18 |
| 175 | Brussels sprout | 0.18 |
| 176 | Histidine | 0.17 |
| 177 | Bearberry | 0.17 |
| 178 | Bitter orange | 0.16 |
| 179 | Barbados Cherry | 0.15 |
| 180 | Proline | 0.14 |
| 181 | Marsh mallow | 0.14 |
| 182 | Hen-of-the-wood | 0.13 |
| 183 | Valine | 0.13 |
| 184 | Hesperidin | 0.12 |
| 185 | Dimethylethanolamine | 0.11 |
| 186 | Hops | 0.11 |
| 187 | Dipeptidyl peptidase-4 | 0.11 |
| 188 | Zeaxanthin | 0.10 |
| 189 | Green coffee | 0.10 |
| 190 | Omega-9 fatty acid | 0.10 |
| 191 | Vanadium | 0.09 |
| 192 | Indian frankincense | 0.09 |
| 193 | Isoleucine | 0.09 |
| 194 | Fructooligosaccharide | 0.09 |
| 195 | gamma-Linolenic acid | 0.08 |
| 196 | Yohimbe | 0.08 |
| 197 | Threonine | 0.06 |
| 198 | Sucrase | 0.03 |
| 199 | Synbiotics | 0.03 |
| 200 | Alpha-galactosidase | 0.02 |

Table S4

The ten most common dietary supplements-related topics in a specific country.
Relative search volume estimated by using adjusted data showing the breakdown by region (Lutein + another dietary supplements-related topic).

| Country | The most common topic |
|---|---|
| Afghanistan | Bitter orange (100), Lutein (50) |
| Albania | Boron (100), Iodine (100), Selenium (100), Silicon (100), Lutein (50) |
| Angola | Passion fruit (100), Yohimbe (100), Lutein (50) |
| Argentina | Magnesium (100), Calcium (99), Cobalamin (99), Ginger (99), Iron (99), Maca (99), Potassium (99), Creatine (98), Vitamin D (98), Aloe vera (97) |
| Armenia | Burdock (100), Dandelion (100), Glycine (100), Hawthorn (100), Hops (100), Lipoic acid (100), Molybdenum (100), Taurine (100), Vitamin B-6 (100), Lutein (50) |
| Australia | Protein (99), Iron (98), Magnesium (98), Vitamin D (97), Caffeine (96), Calcium (96), Cobalamin (95), Vitamin C (95), Fish oil (93), Potassium (93) |
| Austria | Magnesium (99), Vitamin D (97), Cobalamin (95), Iron (94), Calcium (93), Folate (92), Omega-3 fatty acids (92), Potassium (92), Zinc (92), Vitamin C (91) |
| Bahrain | Fennel (100), Fenugreek (100), Olive (100), Parsley (100), Pomegranate (100), Lutein (50) |
| Barbados | Cayenne pepper (100), Lutein (50) |
| Belarus | Magnesium (94), Vitamin B-6 (91), Calcium (88), Vitamin D (88), Folate (87), Iron (87), Glycine (85), Ginger (84), Omega-3 fatty acids (84), Vitamin E (84) |
| Belgium | Magnesium (100), Vitamin D (99), Cobalamin (98), Iron (98), Folate (97), Calcium (96), Omega-3 fatty acids (96), Potassium (96), Aloe vera (95), Creatine (95) |
| Bhutan | Cordyceps (100), Lutein (50) |
| Bosnia & Herzegovina | Amylase (100), Artichoke (100), Astaxanthin (100), beta-Carotene (100), Black pepper (100), Carnitine (100), Chromium (100), Cinnamon (100), Common Nettle (100), Cranberry (100) |
| Brazil | Magnesium (99), Whey protein (97), Ginger (96), Vitamin D (96), Branched-chain amino acid (95), Calcium (95), Creatine (95), Copper (94), Iron (94), Oat (94) |
| Brunei | Alfalfa (100), Mangosteen (100), Lutein (50) |
| Bulgaria | Magnesium (97), Carnitine (91), Vitamin D (91), Aloe vera (90), Cobalamin (88), Creatine (88), Probiotic (88), Selenium (87), Omega-3 fatty acids (86), Calcium (85) |
| Cambodia | Mangosteen (100), Lutein (50) |
| Cameroon | Glutathione (100), Parsley (100), Lutein (50) |
| Canada | Protein (98), Magnesium (97), Iron (96), Caffeine (95), Calcium (95), Cobalamin (95), Vitamin D (95), Omega-3 fatty acids (92), Potassium (92), Vitamin C (91) |
| Chile | Magnesium (100), Calcium (99), Ginger (99), Iron (99), Omega-3 fatty acids (99), Potassium (99), Spirulina (99), Vitamin D (99), Aloe vera (98), Cobalamin (98) |
| China | Calcium (98), Iron (93), Zinc (93), Vitamin C (92), Potassium (91), Iodine (90), Maidenhair tree (90), Vitamin E (90), Ginger (89), Spirulina (89) |
| Colombia | Magnesium (99), Aloe vera (98), Calcium (98), Ginger (98), Potassium (98), Iron (97), Methylsulfonylmethane (97), Thiamine (97), Vitamin C (97), Cobalamin (96) |
| Côte d’Ivoire | Glutathione (100), Lutein (50) |
| Croatia | Iron (99), Magnesium (99), Ginger (97), Garlic (96), Maca (96), Olive (96), Potassium (96), Probiotic (96), Vitamin C (96), Vitamin D (96) |
| Cuba | Chondroitin sulfate (100), Lutein (50) |
| Czechia | Magnesium (96), Iron (91), Calcium (89), Vitamin C (89), Garlic (88), Ginger (88), Vitamin D (88), Milk thistle (87), Potassium (87), Branched-chain amino acid (85) |
| Denmark | Magnesium (99), Vitamin D (99), Cobalamin (97), Ginger (97), Calcium (96), Fish oil (96), Iron (96), Potassium (96), Aloe vera (95), Creatine (95) |

| | |
|---|---|
| Egypt | Calcium (99), Ginger (99), Iron (99), Vitamin D (99), Cinnamon (98), Cobalamin (98), Fenugreek (98), Garlic (98), Magnesium (98), Olive (98) |
| Finland | Magnesium (100), Iron (99), Vitamin D (99), Calcium (98), Cobalamin (98), Melatonin (98), Zinc (98), Methylsulfonylmethane (97), Omega-3 fatty acids (97), Potassium (97) |
| France | Iron (99), Magnesium (99), Vitamin D (98), Aloe vera (97), Copper (97), Ginger (97), Potassium (97), Calcium (96), Folate (96), Probiotic (96) |
| Germany | Magnesium (99), Vitamin D (97), Cobalamin (96), Iron (94), Calcium (92), Potassium (92), Folate (91), Zinc (91), Omega-3 fatty acids (90), Maidenhair tree (87) |
| Ghana | Aloe vera (100), Beetroot (100), Bell pepper (100), Black pepper (100), Cayenne pepper (100), Centella asiatica (100), Cinnamon (100), Cordyceps (100), Dandelion (100), Female ginseng (100) |
| Greece | Magnesium (99), Aloe vera (98), Iron (98), Vitamin D (98), Cobalamin (96), Omega-3 fatty acids (96), Protein (96), Calcium (95), Creatine (95), Folate (95) |
| Honduras | Aloe vera (100), Bitter orange (100), Conjugated linoleic acid (100), Field horsetail (100), Flax seed (100), Hesperidin (100), Isoleucine (100), Lecithin (100), Oregano (100), Papaya (100) |
| Hong Kong | Calcium (91), Vitamin C (87), Potassium (85), Probiotic (83), Protein (83), Iron (82), Glucosamine (80), Folate (79), Vitamin D (79), Ginger (78) |
| Hungary | Magnesium (99), Vitamin D (92), Calcium (91), Vitamin B-6 (91), Iron (90), Aloe vera (87), Garlic (87), Potassium (87), Omega-3 fatty acids (86), Creatine (85) |
| Iceland | Astaxanthin (100), Lutein (50) |
| India | Protein (100), Calcium (98), Magnesium (98), Vitamin D (98), Aloe vera (97), Cobalamin (97), Iron (97), Whey protein (97), Folate (96), Potassium (96) |
| Indonesia | Garlic (99), Ginger (99), Magnesium (99), Papaya (99), Aloe vera (97), Calcium (97), Mangosteen (97), Green tea (96), Olive (96), Vitamin C (96) |
| Iran | Calcium (100), Fennel (100), Garlic (100), Iron (100), Vitamin D (100), Zinc (100), Cinnamon (99), Creatine (99), Flax seed (99), Folate (99) |
| Ireland | Protein (98), Magnesium (97), Iron (96), Vitamin D (96), Cobalamin (95), Calcium (94), Caffeine (92), Aloe vera (91), Creatine (91), Vitamin C (91) |
| Israel | Iron (99), Cobalamin (98), Magnesium (98), Vitamin D (98), Calcium (97), Omega-3 fatty acids (97), Folate (96), Potassium (96), Probiotic (95), Garlic (94) |
| Italy | Magnesium (99), Aloe vera (95), Iron (95), Vitamin D (95), Ginger (94), Potassium (94), Cobalamin (92), Folate (92), Omega-3 fatty acids (91), Acetylcysteine (90) |
| Japan | Iron (90), Folate (86), Zinc (86), Potassium (84), Calcium (82), Vitamin C (82), Caffeine (77), Dietary fiber (77), Garlic (77), Magnesium (77) |
| Kazakhstan | Magnesium (95), Calcium (94), Vitamin B-6 (93), Aloe vera (92), Folate (91), Vitamin D (91), Ginger (90), Vitamin E (90), Iron (89), Acetylcysteine (87) |
| Kenya | Aloe vera (100), Ashwagandha (100), Beetroot (100), Bell pepper (100), beta-Carotene (100), Black pepper (100), Calcium (100), Cayenne pepper (100), Chlorophyll (100), Cinnamon (100) |
| Kosovo | Iodine (100), Maca (100), Selenium (100), Silicon (100), Lutein (50) |
| Libya | Cinnamon (100), Fennel (100), Fenugreek (100), Ginger (100), Olive (100), Parsley (100), Pomegranate (100), Rosemary (100), Vitamin D (100), Lutein (50) |
| Lithuania | Magnesium (99), Iron (96), Vitamin D (95), Potassium (94), Calcium (93), Vitamin C (91), Creatine (90), Garlic (90), Folate (89), Ginger (89) |
| Malaysia | Vitamin C (95), Protein (93), Calcium (91), Garlic (91), Aloe vera (89), Magnesium (89), Fish oil (87), Folate (87), Iron (87), Ginger (85) |
| Mauritius | Holy Basil (100), Lutein (50) |
| Mexico | Magnesium (99), Calcium (98), Aloe vera (97), Iron (97), Potassium (97), Carnitine (96), Garlic (96), Ginger (96), Folate (95), Omega-3 fatty acids (95) |
| Montenegro | Goji (100), Hops (100), Marsh mallow (100), Red raspberry (100), Selenium (100), Silicon (100), Lutein (50) |

| | |
|---|---|
| Mozambique | Yohimbe (100), Lutein (50) |
| Myanmar (Burma) | Mangosteen (100), Velvet bean (100), Lutein (50) |
| Nepal | Ashwagandha (100), Cordyceps (100), Holy Basil (100), Indian gooseberry (100), Lutein (50) |
| Netherlands | Cobalamin (99), Magnesium (99), Vitamin D (99), Melatonin (97), Calcium (96), Folate (96), Potassium (96), Vitamin C (96), Creatine (95), Iron (95) |
| New Zealand | Protein (99), Iron (97), Magnesium (97), Vitamin C (96), Caffeine (95), Calcium (95), Cobalamin (95), Vitamin D (95), Potassium (93), Zinc (92) |
| Nicaragua | Bitter orange (100), Field horsetail (100), Flax seed (100), Glutamic acid (100), Hesperidin (100), Lecithin (100), Papaya (100), Phosphorus (100), Lutein (50) |
| North Macedonia | Barbados Cherry (100), beta-Carotene (100), Common Nettle (100), Iodine (100), Maca (100), Marsh mallow (100), Oregano (100), Selenium (100), Silicon (100), Spirulina (100) |
| Norway | Vitamin D (99), Cobalamin (98), Iron (98), Magnesium (98), Calcium (97), Creatine (97), Ginger (97), Omega-3 fatty acids (97), Caffeine (96), Folate (96) |
| Pakistan | Protein (99), Vitamin D (99), Calcium (98), Magnesium (98), Iron (97), Aloe vera (96), Folate (96), Cobalamin (95), Potassium (95), Vitamin C (95) |
| Palestine | Cinnamon (100), Fennel (100), Fenugreek (100), Hawthorn (100), Olive (100), Parsley (100), Pomegranate (100), Rosemary (100), Lutein (50) |
| Paraguay | Amylase (100), Barbados Cherry (100), Betaine (100), Bitter orange (100), Citrulline (100), Conjugated linoleic acid (100), Copper (100), Creatine (100), Field horsetail (100), Garlic (100) |
| Peru | Magnesium (99), Calcium (98), Iron (98), Aloe vera (97), Maca (97), Methylsulfonylmethane (97), Potassium (97), Vitamin C (97), Cobalamin (96), Folate (95) |
| Philippines | Glutathione (97), Protein (97), Vitamin C (97), Calcium (95), Iodine (95), Iron (95), Magnesium (95), Potassium (94), Vitamin E (94), Turmeric (93) |
| Poland | Magnesium (84), Iron (72), Vitamin D (72), Calcium (67), Creatine (67), Potassium (67), Cobalamin (59), Garlic (58), Vitamin B-6 (53), Dietary fiber (51) |
| Portugal | Magnesium (100), Ginger (98), Oat (98), Iron (97), Vitamin D (97), Calcium (96), Cobalamin (96), Potassium (96), Cinnamon (94), Creatine (94) |
| Réunion | Barbados Cherry (100), Betaine (100), Lutein (50) |
| Romania | Magnesium (98), Calcium (94), Iron (91), Vitamin C (90), Omega-3 fatty acids (89), Vitamin D (88), Vitamin B-6 (87), Copper (85), Folate (84), Aloe vera (82) |
| Russia | Magnesium (94), Vitamin B-6 (93), Calcium (91), Carnitine (91), Vitamin D (91), Iron (90), Folate (89), Vitamin E (89), Glycine (88), Ginger (87) |
| Saudi Arabia | Vitamin D (100), Fenugreek (99), Ginger (99), Iron (99), Calcium (98), Cinnamon (98), Garlic (98), Magnesium (98), Olive (98), Pomegranate (98) |
| Senegal | Glutathione (100), Lutein (50) |
| Serbia | Magnesium (99), Iron (98), Selenium (98), Ginger (97), Iodine (97), Garlic (96), Probiotic (96), Vitamin C (96), Vitamin D (96), Zinc (96) |
| Singapore | Protein (94), Calcium (89), Vitamin C (88), Iron (86), Magnesium (85), Potassium (84), Caffeine (83), Vitamin D (83), Fish oil (82), Omega-3 fatty acids (82) |
| Slovakia | Magnesium (98), Iron (93), Calcium (92), Vitamin C (91), Creatine (90), Vitamin D (90), Probiotic (89), Aloe vera (88), Vitamin B-6 (87), Zinc (87) |
| South Africa | Aloe vera (99), Iron (99), Magnesium (99), Protein (99), Calcium (98), Folate (98), Ginger (98), Vitamin C (98), Vitamin D (98), Caffeine (97) |
| South Korea | Calcium (87), Vitamin C (87), Omega-3 fatty acids (85), Vitamin D (84), Magnesium (82), Potassium (77), Five-flavor berry (75), Iron (75), Zinc (75), Caffeine (73) |
| Spain | Magnesium (99), Iron (98), Calcium (97), Cobalamin (97), Potassium (97), Vitamin D (97), Maca (96), Ginger (95), Omega-3 fatty acids (95), Vitamin C (95) |
| Sri Lanka | 4-Aminobenzoic acid (100), alpha-Linolenic acid (100), Amylase (100), Ashwagandha (100), Centella asiatica (100), Fenugreek (100), Fish oil (100), Glutathione (100), Histidine |

| | |
|---|---|
| | (100), Indian gooseberry (100) |
| Sudan | Cinnamon (100), Fennel (100), Fenugreek (100), Garlic (100), Ginger (100), Olive (100), Parsley (100), Pomegranate (100), Lutein (50) |
| Sweden | Magnesium (99), Vitamin D (98), Cobalamin (97), Omega-3 fatty acids (97), Iron (96), Calcium (95), Creatine (95), Folate (95), Potassium (95), Vitamin C (95) |
| Switzerland | Magnesium (99), Cobalamin (97), Vitamin D (97), Iron (96), Calcium (93), Omega-3 fatty acids (93), Potassium (93), Folate (92), Ginger (91), Zinc (91) |
| Syria | Bitter orange (100), Cinnamon (100), Common Nettle (100), Fennel (100), Fenugreek (100), Flax seed (100), Garlic (100), Ginger (100), Hawthorn (100), Lemon balm (100) |
| Taiwan | Calcium (62), Probiotic (58), Lutein (50), Fish oil (46), Vitamin C (45), Iron (42), Potassium (39), Turmeric (39), Vitamin D (33), Folate (32) |
| Tanzania | Nickel (100), Lutein (50) |
| Thailand | Glutathione (98), Vitamin C (98), Aloe vera (95), Turmeric (94), Calcium (93), Iron (93), Ginger (89), Vitamin D (88), Magnesium (85), Garlic (84) |
| Tunisia | Aloe vera (100), Artichoke (100), Barbados Cherry (100), Bearberry (100), Burdock (100), Carnitine (100), Cinnamon (100), Conjugated linoleic acid (100), Fennel (100), Fenugreek (100) |
| Turkey | Magnesium (100), Cobalamin (99), Garlic (99), Ginger (99), Iron (99), Vitamin D (99), Cinnamon (98), Parsley (98), Calcium (97), Fish oil (97) |
| Turkmenistan | Glycine (100), Lutein (50) |
| Uganda | Beetroot (100), Lutein (50) |
| Ukraine | Magnesium (93), Calcium (90), Vitamin B-6 (90), Vitamin E (85), Vitamin D (84), Ginger (83), Acetylcysteine (82), Iodine (82), Creatine (81), Folate (81) |
| United Arab Emirates | Protein (98), Vitamin D (98), Iron (97), Magnesium (97), Calcium (96), Ginger (96), Vitamin C (95), Cobalamin (94), Fenugreek (94), Garlic (94) |
| United Kingdom | Protein (99), Vitamin D (98), Cobalamin (97), Iron (97), Caffeine (96), Calcium (96), Magnesium (96), Aloe vera (94), Creatine (93), Folate (93) |
| United States | Protein (99), Magnesium (98), Caffeine (97), Vitamin D (97), Calcium (96), Iron (96), Cobalamin (95), Potassium (95), Dietary fiber (94), Probiotic (92) |
| Venezuela | Magnesium (99), Potassium (99), Aloe vera (98), Calcium (97), Folate (96), Carnitine (95), Cobalamin (95), Creatine (95), Iron (95), Methylsulfonylmethane (95) |
| Vietnam | Calcium (100), Iron (100), Garlic (99), Zinc (99), Cinnamon (98), Copper (98), Ginger (98), Magnesium (98), Turmeric (98), Vitamin E (97) |
| Yemen | Fennel (100), Fenugreek (100), Garlic (100), Olive (100), Pomegranate (100), Lutein (50) |
| Zimbabwe | Cayenne pepper (100), Lutein (50) |

Table S5
Time series analysis of n = 200 topics related to dietary suplements.

| Topic | Seasonal Mann-Kendall test | Slope [RSV/year] | TBATS (seasonality present, period [month]) | Month with the highest seasonal component [RSV] | Month with the lowest seasonal component [RSV] | Seasonal component amplitude [RSV] |
|---|---|---|---|---|---|---|
| 4-Aminobenzoic acid | tau = -0.84; *** | -3.37; *** | YES, 12 | April (7.06) | December (-10.15) | 17.21 |
| 5-Hydroxytryptophan | tau = -0.28; *** | -0.90; *** | YES, 12 | August (3.99) | December (-7.19) | 11.18 |
| Acai berry | tau = 0.01; 0.803 | - | YES, 12 | January (4.56) | June (-2.78) | 7.34 |
| Acetylcarnitine | tau = 0.07; 0.196 | - | YES, 12 | March (4.01) | December (-7.81) | 11.83 |
| Acetylcysteine | tau = 0.93; *** | 3.45; *** | YES, 12 | January (8.94) | August (-8.54) | 17.48 |
| Alanine | tau = -0.49; *** | -1.36; *** | YES, 12 | April (7.76) | August (-10.65) | 18.41 |
| Alfalfa | tau = 0.46; *** | 1.57; *** | YES, 12 | April (7.13) | December (-8.44) | 15.57 |
| Aloe vera | tau = 0.75; *** | 3.48; *** | YES, 12 | July (4.58) | December (-8.81) | 13.39 |
| Alpha-galactosidase | tau = -0.21; *** | -0.81; *** | NO, - | - | - | - |
| alpha-Linolenic acid | tau = -0.76; *** | -2.54; *** | YES, 12 | March (5.18) | August (-5.49) | 10.67 |
| Amylase | tau = 0.06; 0.286 | - | YES, 12 | November (9.46) | July (-15.76) | 25.23 |
| Arginine | tau = 0.18; ** | 0.27; ** | YES, 12 | February (5.31) | December (-7.91) | 13.21 |
| Artichoke | tau = 0.44; *** | 2.11; *** | YES, 12 | April (14.49) | December (-13.09) | 27.58 |
| Ashwagandha | tau = 0.82; *** | 4.25; *** | YES, 12 | March (1.54) | December (-2.78) | 4.32 |
| Astaxanthin | tau = 0.70; *** | 4.41; *** | YES, 12 | June (4.06) | December (-7.63) | 11.69 |
| Barbados Cherry | tau = 0.73; *** | 2.40; *** | YES, 12 | February (9.94) | July (-8.49) | 18.42 |
| Bearberry | tau = -0.37; *** | -0.97; *** | YES, 12 | August (4.00) | December (-4.27) | 8.27 |
| Beetroot | tau = 0.94; *** | 5.42; *** | YES, 12 | January (4.52) | December (-7.38) | 11.91 |
| Bell pepper | tau = 0.95; *** | 4.69; *** | YES, 12 | August (6.24) | December (-16.16) | 22.40 |
| beta-Carotene | tau = -0.82; *** | -2.36; *** | YES, 12 | May (7.95) | December (-7.69) | 15.65 |
| Betaine | tau = 0.42; *** | 1.49; *** | NO, - | - | - | - |
| Bilberry | tau = -0.21; *** | -0.98; *** | YES, 12 | July (8.16) | December (-7.36) | 15.53 |
| Biotin | tau = 0.27; *** | 0.94; *** | YES, 12 | February (1.98) | December (-5.52) | 7.50 |
| Bitter orange | tau = 0.35; *** | 0.77; *** | YES, 12 | January (7.00) | September (-6.48) | 13.48 |
| Black cohosh | tau = -0.75; *** | -2.59; *** | YES, 12 | August (5.86) | December (-10.92) | 16.78 |
| Black pepper | tau = 0.95; *** | 3.09; *** | YES, 12 | May (1.86) | June (-2.75) | 4.61 |
| Blackberry | tau = 0.89; *** | 4.26; *** | YES, 12 | August (14.84) | December (-10.93) | 25.76 |

| | | | | | | |
|---|---|---|---|---|---|---|
| Blackcurrant | tau = 0.68; *** | 1.59; *** | YES, 12 | July (12.25) | December (-7.12) | 19.36 |
| Blueberry | tau = 0.79; *** | 2.32; *** | YES, 12 | July (24.11) | December (-16.49) | 40.59 |
| Boron | tau = 0.63; *** | 2.32; *** | YES, 12 | July (4.31) | December (-6.49) | 10.80 |
| Branched-chain amino acid | tau = 0.86; *** | 5.45; *** | YES, 12 | March (3.65) | December (-9.51) | 13.16 |
| Broccoli | tau = 0.84; *** | 3.38; *** | YES, 12 | January (12.22) | December (-9.96) | 22.19 |
| Bromelain | tau = 0.13; 0.016 | 0.21; 0.234 | YES, 12 | February (5.87) | December (-7.72) | 13.59 |
| Brussels sprout | tau = 0.92; *** | 3.81; *** | YES, 12 | January (16.59) | August (-12.19) | 28.78 |
| Burdock | tau = 0.64; *** | 1.97; *** | YES, 12 | May (11.19) | December (-6.84) | 18.03 |
| Caffeine | tau = 0.48; *** | 1.21; *** | YES, 12 | October (4.93) | December (-6.40) | 11.33 |
| Calcium | tau = 0.16; ** | 0.41; ** | YES, 12 | April (4.91) | December (-10.41) | 15.32 |
| Carnitine | tau = -0.08; 0.159 | - | YES, 12 | April (8.82) | December (-17.64) | 26.46 |
| Cayenne pepper | tau = 0.40; *** | 1.78; *** | YES, 12 | January (13.26) | December (-7.95) | 21.21 |
| Centella asiatica | tau = 0.50; *** | 0.88; *** | YES, 12 | August (5.28) | December (-9.29) | 14.57 |
| Chlorella | tau = 0.16; ** | -0.19; 0.265 | YES, 12 | March (4.15) | December (-9.23) | 13.38 |
| Chlorophyll | tau = 0.69; *** | 3.07; *** | YES, 12 | March (5.31) | December (-6.91) | 12.22 |
| Choline | tau = -0.30; *** | -1.20; *** | YES, 12 | April (2.19) | December (-4.40) | 6.59 |
| Chondroitin sulfate | tau = -0.02; 0.772 | - | YES, 12 | March (3.16) | December (-7.07) | 10.23 |
| Chromium | tau = -0.59; *** | -0.40; ** | YES, 12 | April (6.57) | December (-2.93) | 9.49 |
| Cinnamon | tau = 0.90; *** | 5.42; *** | YES, 12 | January (6.59) | June (-5.98) | 12.56 |
| Citrulline | tau = 0.80; *** | 4.55; *** | YES, 12 | July (4.31) | December (-6.52) | 10.83 |
| Cobalamin | tau = 0.94; *** | 3.12; *** | YES, 12 | March (2.77) | December (-7.21) | 9.98 |
| Common Nettle | tau = 0.65; *** | 1.34; *** | YES, 12 | May (19.97) | December (-17.02) | 36.99 |
| Coneflower | tau = -0.12; 0.03 | -0.57; ** | YES, 12 | October (13.01) | June (-11.57) | 24.59 |
| Conjugated linoleic acid | tau = -0.44; *** | -1.57; *** | YES, 12 | May (6.00) | December (-14.66) | 20.66 |
| Copper | tau = 0.86; *** | 2.92; *** | YES, 12 | October (4.12) | December (-6.49) | 10.61 |
| Cordyceps | tau = 0.59; *** | 1.66; *** | NO, - | - | - | - |
| Cranberry | tau = 0.91; *** | 3.11; *** | YES, 12 | November (9.89) | June (-7.72) | 17.61 |
| Creatine | tau = -0.64; *** | -1.57; *** | YES, 12 | February (5.72) | December (-11.08) | 16.80 |
| Curly kale | tau = 0.63; *** | 4.58; *** | YES, 12 | January (9.53) | December (-5.97) | 15.49 |
| Cysteine | tau = -0.51; *** | -1.87; *** | YES, 12 | October (4.99) | December (-4.12) | 9.11 |
| Dandelion | tau = 0.70; *** | 2.22; *** | YES, 12 | May (25.02) | December (-12.15) | 37.17 |
| Dietary fiber | tau = 0.55; *** | 1.14; *** | YES, 12 | March (7.98) | December (-15.17) | 23.15 |
| Dimethylethanolamine | tau = -0.71; *** | -2.45; *** | YES, 12 | February (2.81) | December (-6.09) | 8.89 |
| Dipeptidyl peptidase-4 | tau = 0.49; *** | 0.89; *** | NO, - | - | - | - |

| | | | | | | |
|---|---|---|---|---|---|---|
| Docosahexaenoic acid | tau = 0.79; *** | 2.74; *** | YES, 12 | October (3.31) | December (-7.24) | 10.55 |
| Eicosapentaenoic acid | tau = 0.13; 0.017 | 0.21; 0.208 | YES, 12 | February (4.79) | December (-8.43) | 13.22 |
| Female ginseng | tau = -0.84; *** | -3.70; *** | NO, - | - | - | - |
| Fennel | tau = 0.96; *** | 4.87; *** | YES, 12 | January (4.54) | June (-4.25) | 8.79 |
| Fenugreek | tau = 0.89; *** | 4.81; *** | YES, 12 | February (2.71) | December (-3.33) | 6.04 |
| Field horsetail | tau = 0.70; *** | 2.23; *** | YES, 12 | May (14.76) | December (-17.49) | 32.26 |
| Fish oil | tau = 0.54; *** | 1.01; *** | YES, 12 | January (8.27) | December (-7.41) | 15.68 |
| Five-flavor berry | tau = -0.36; *** | -0.69; *** | YES, 12 | April (5.08) | December (-6.42) | 11.50 |
| Flavonoid | tau = -0.88; *** | -3.05; *** | YES, 12 | March (3.86) | December (-3.71) | 7.57 |
| Flax seed | tau = 0.24; *** | 0.50; ** | YES, 12 | February (7.51) | December (-14.29) | 21.80 |
| Folate | tau = 0.55; *** | 1.95; *** | YES, 12 | March (2.56) | December (-9.16) | 11.72 |
| Fructooligosaccharide | tau = -0.05; 0.385 | - | YES, 12 | March (5.50) | December (-9.51) | 15.01 |
| gamma-Aminobutyric acid | tau = -0.14; 0.012 | -0.67; *** | YES, 12 | April (5.38) | December (-6.56) | 11.95 |
| gamma-Linolenic acid | tau = -0.63; *** | -2.13; *** | NO, - | - | - | - |
| Garcinia cambogia | tau = 0.31; *** | 2.38; *** | YES, 12 | April (3.53) | December (-4.22) | 7.74 |
| Garlic | tau = 0.90; *** | 5.07; *** | YES, 12 | April (2.99) | June (-3.20) | 6.19 |
| Ginger | tau = 0.89; *** | 4.93; *** | YES, 12 | January (9.38) | June (-5.86) | 15.23 |
| Glucosamine | tau = -0.46; *** | -2.24; *** | YES, 12 | April (2.19) | December (-7.39) | 9.58 |
| Glutamic acid | tau = -0.69; *** | -1.57; *** | YES, 12 | May (4.99) | August (-6.27) | 11.26 |
| Glutamine | tau = 0.61; *** | 2.08; *** | YES, 12 | April (4.52) | December (-12.07) | 16.59 |
| Glutathione | tau = 0.72; *** | 2.88; *** | YES, 12 | June (3.75) | December (-6.67) | 10.43 |
| Glycine | tau = 0.83; *** | 3.32; *** | YES, 12 | March (5.61) | July (-7.93) | 13.53 |
| Goji | tau = 0.41; *** | 1.73; *** | YES, 12 | March (6.00) | December (-5.25) | 11.26 |
| Goldenseal | tau = -0.84; *** | -2.28; *** | YES, 12 | October (2.66) | June (-4.11) | 6.76 |
| Grape seed extract | tau = -0.81; *** | -2.39; *** | YES, 12 | May (2.63)- | November (-3.36) | 5.99 |
| Green coffee | tau = 0.54; *** | 1.02; *** | NO, - | - | - | - |
| Green tea | tau = 0.70; *** | 2.54; *** | YES, 12 | January (10.20) | December (-9.11) | 19.31 |
| Guarana | tau = -0.71; *** | -1.69; *** | YES, 12 | April (6.03) | December (-12.44) | 18.47 |
| Hawthorn | tau = 0.37; *** | 0.67; *** | YES, 12 | October (7.78) | July (-6.41) | 14.19 |
| Hen-of-the-wood | tau = 0.32; *** | 0.60; ** | YES, 12 | October (23.29) | December (-6.48) | 29.77 |
| Hesperidin | tau = 0.69; *** | 3.11; *** | YES, 12 | July (2.09) | December (-3.46) | 5.54 |
| Histidine | tau = -0.58; *** | -1.49; *** | YES, 12 | October (10.20) | July (-12.41) | 22.61 |
| Holy Basil | tau = 0.77; *** | 3.88; *** | YES, 12 | September (5.46) | May (-5.46) | 10.92 |
| Hops | tau = 0.31; *** | 0.48; ** | YES, 12 | September (13.28) | December (-12.55) | 25.84 |

| | | | | | | |
|---|---|---|---|---|---|---|
| Hyaluronic acid | tau = 0.72; *** | 2.74; *** | YES, 12 | March (4.44) | December (-5.58) | 10.02 |
| Indian frankincense | tau = 0.65; *** | 1.99; *** | NO, - | - | - | - |
| Indian gooseberry | tau = 0.89; *** | 4.25; *** | YES, 12 | November (5.64) | May (-6.28) | 11.92 |
| Inositol | tau = 0.06; 0.242 | - | NO, - | - | - | - |
| Iodine | tau = 0.53; *** | 0.39; *** | YES, 12 | March (5.93) | December (-3.92) | 9.84 |
| Iron | tau = 0.93; *** | 2.45; *** | YES, 12 | March (3.83) | December (-9.04) | 12.88 |
| Isoleucine | tau = -0.07; 0.205 | - | YES, 12 | October (6.96) | December (-8.64) | 15.60 |
| Kelp | tau = -0.55; *** | -0.84; *** | YES, 12 | March (6.15) | December (-7.61) | 13.76 |
| Lactase | tau = 0.48; *** | 1.42; *** | YES, 12 | October (7.85) | July (-8.27) | 16.12 |
| Lecithin | tau = -0.88; *** | -2.18; *** | YES, 12 | January (3.15) | December (-6.00) | 9.16 |
| Lemon balm | tau = 0.93; *** | 3.66; *** | YES, 12 | May (12.19) | December (-10.68) | 22.87 |
| Leucine | tau = 0.36; *** | 0.57; *** | YES, 12 | October (4.00) | July (-6.37) | 10.38 |
| Lingzhi mushroom | tau = 0.39; *** | 0.91; *** | YES, 12 | February (2.34) | December (-4.14) | 6.48 |
| Linoleic acid | tau = -0.85; *** | -3.01; *** | YES, 12 | April (7.10) | December (-7.77) | 14.86 |
| Linseed oil | tau = -0.48; *** | -1.64; *** | YES, 12 | January (7.77) | December (-8.02) | 15.79 |
| Lipase | tau = 0.10; 0.067 | - | YES, 12 | April (6.69) | August (-9.78) | 16.47 |
| Lipoic acid | tau = 0.00; 0.989 | - | YES, 12 | February (5.02) | December (-2.13) | 7.15 |
| Liquorice | tau = 0.78; *** | 2.82; *** | YES, 12 | January (4.64) | August (-3.03) | 7.67 |
| Lutein | tau = -0.04; 0.486 | - | YES, 12 | March (3.99) | December (-5.97) | 9.96 |
| Lycopene | tau = -0.53; *** | -1.81; *** | YES, 12 | August (3.19) | December (-4.04) | 7.23 |
| Lysine | tau = 0.28; *** | 0.36; *** | YES, 12 | February (3.72) | July (-3.09) | 6.81 |
| Maca | tau = 0.73; *** | 3.12; *** | YES, 12 | February (2.65) | December (-8.17) | 10.83 |
| Magnesium | tau = 0.94; *** | 4.60; *** | YES, 12 | March (2.56) | December (-5.22) | 7.78 |
| Maidenhair tree | tau = -0.54; *** | -1.99; *** | YES, 12 | November (8.33) | December (-4.62) | 12.95 |
| Manganese | tau = -0.45; *** | -1.34; *** | YES, 12 | March (5.58) | December (-8.03) | 13.61 |
| Mangosteen | tau = -0.26; *** | -1.31; *** | YES, 12 | July (6.45) | December (-8.63) | 15.09 |
| Marsh mallow | tau = 0.85; *** | 4.43; *** | YES, 12 | January (12.26) | July (-11.88) | 24.14 |
| Melatonin | tau = 0.82; *** | 2.97; *** | YES, 12 | January (5.96) | December (-4.47) | 10.44 |
| Methionine | tau = -0.68; *** | -1.63; *** | YES, 12 | March (4.22) | December (-6.41) | 10.64 |
| Methylsulfonylmethane | tau = -0.63; *** | -3.60; *** | YES, 12 | February (1.89) | December (-4.96) | 6.85 |
| Milk thistle | tau = 0.68; *** | 2.04; *** | YES, 12 | January (6.79) | December (-6.34) | 13.13 |
| Milkvetch | tau = -0.62; *** | -2.04; *** | YES, 12 | November (4.06) | July (-4.75) | 8.82 |
| Molybdenum | tau = -0.75; *** | -2.40; *** | YES, 12 | April (4.68) | July (-5.84) | 10.51 |
| Monounsaturated fat | tau = -0.27; *** | -0.33; ** | YES, 12 | February (5.65) | December (-8.00) | 13.65 |

| | | | | | | |
|---|---|---|---|---|---|---|
| Niacin | tau = -0.61; *** | -1.14; *** | YES, 12 | April (4.71) | December (-7.20) | 11.91 |
| Nickel | tau = 0.10; 0.058 | - | YES, 12 | May (7.19) | December (-6.81) | 13.99 |
| Oat | tau = 0.94; *** | 4.94; *** | YES, 12 | February (3.31) | December (-9.56) | 12.87 |
| Oleic acid | tau = -0.75; *** | -2.31; *** | YES, 12 | May (7.31) | August (-10.51) | 17.83 |
| Olive | tau = 0.9; *** | 4.50; *** | YES, 12 | February (4.46) | December (-3.34) | 7.80 |
| Omega-3 fatty acids | tau = 0.36; *** | 1.12; *** | YES, 12 | March (6.30) | December (-10.50) | 16.79 |
| Omega-6 fatty acid | tau = -0.63; *** | -1.33; *** | YES, 12 | March (8.49) | December (-13.77) | 22.26 |
| Omega-9 fatty acid | tau = -0.55; *** | -1.37; *** | YES, 12 | April (3.42) | December (-7.90) | 11.32 |
| Oregano | tau = 0.82; *** | 3.75; *** | YES, 12 | January (6.70) | August (-4.06) | 10.76 |
| Pantothenic acid | tau = -0.50; *** | -1.23; *** | YES, 12 | April (5.95) | December (-8.76) | 14.71 |
| Papain | tau = -0.57; *** | -2.34; *** | YES, 12 | March (3.68) | December (-6.43) | 10.12 |
| Papaya | tau = 0.84; *** | 5.21; *** | YES, 12 | March (3.37) | December (-7.41) | 10.77 |
| Parsley | tau = 0.89; *** | 5.40; *** | YES, 12 | March (5.70) | December (-7.12) | 12.82 |
| Passion fruit | tau = 0.51; *** | 2.96; *** | NO, - | - | - | - |
| Pectin | tau = 0.30; *** | 0.40; 0.011 | YES, 12 | May (3.69) | December (-4.66) | 8.36 |
| Peppermint | tau = 0.83; *** | 4.29; *** | YES, 12 | January (2.95) | August (-2.13) | 5.08 |
| Phenylalanine | tau = -0.87; *** | -3.33; *** | YES, 12 | May (4.04) | December (-9.62) | 13.66 |
| Phosphatidylcholine | tau = -0.85; *** | -3.11; *** | YES, 12 | April (5.75) | December (-7.97) | 13.72 |
| Phosphatidylserine | tau = -0.80; *** | -2.15; *** | YES, 12 | September (1.56) | December (-4.56) | 6.12 |
| Phosphorus | tau = -0.26; *** | -0.81; *** | YES, 12 | October (6.91) | December (-8.72) | 15.62 |
| Polyunsaturated fat | tau = -0.5; *** | -1.21; *** | YES, 12 | October (10.69) | July (-12.13) | 22.81 |
| Pomegranate | tau = 0.89; *** | 3.27; *** | YES, 12 | November (25.38) | June (-12.43) | 37.80 |
| Potassium | tau = 0.69; *** | 1.45; *** | YES, 12 | March (5.47) | December (-10.44) | 15.90 |
| Prebiotic | tau = 0.77; *** | 2.74; *** | YES, 12 | May (4.37) | December (-6.70) | 11.07 |
| Probiotic | tau = 0.98; *** | 5.07; *** | YES, 12 | January (3.87) | December (-4.59) | 8.46 |
| Proline | tau = -0.48; *** | -1.36; *** | YES, 12 | October (5.68) | July (-6.74) | 12.42 |
| Protease | tau = -0.94; *** | -3.44; *** | YES, 12 | November (4.98) | August (-7.92) | 12.90 |
| Protein | tau = 0.68; *** | 1.78; *** | YES, 12 | April (6.77) | December (-14.23) | 21.00 |
| Quercetin | tau = -0.28; *** | -0.89; *** | YES, 12 | April (8.89) | December (-7.19) | 16.09 |
| Raspberry | tau = 0.42; *** | 1.40; *** | YES, 12 | February (4.50) | December (-6.35) | 10.85 |
| Red Clover | tau = -0.59; *** | -1.04; *** | YES, 12 | May (3.82) | December (-7.86) | 11.68 |
| Red raspberry | tau = 0.86; *** | 3.75; *** | YES, 12 | July (17.83) | December (-9.73) | 27.56 |
| Resveratrol | tau = 0.20; *** | 0.70; *** | NO, - | - | - | - |
| Rhodiola rosea | tau = 0.40; *** | 0.86; *** | YES, 12 | February (4.20) | December (-10.91) | 15.11 |

| | | | | | | |
|---|---|---|---|---|---|---|
| Riboflavin | tau = -0.04; 0.477 | - | YES, 12 | November (7.71) | December (-9.46) | 17.17 |
| Rose hip | tau = 0.76; *** | 2.79; *** | YES, 12 | October (11.96) | June (-10.68) | 22.64 |
| Rosemary | tau = 0.90; *** | 4.12; *** | YES, 12 | May (5.62) | December (-5.76) | 11.38 |
| Rutin | tau = -0.42; *** | -1.48; *** | NO, - | - | - | - |
| Saturated fat | tau = 0.48; *** | 0.94; *** | YES, 12 | October (11.00) | July (-16.06) | 27.07 |
| Saw palmetto extract | tau = -0.76; *** | -2.63; *** | YES, 12 | February (3.19) | May (-2.27) | 5.46 |
| Selenium | tau = 0.42; *** | 1.02; *** | YES, 12 | March (4.70) | December (-8.62) | 13.32 |
| Siberian Ginseng | tau = -0.89; *** | -2.43; *** | YES, 12 | October (5.10) | December (-4.46) | 9.56 |
| Silicon | tau = 0.05; 0.392 | - | YES, 12 | April (3.71) | December (-6.61) | 10.32 |
| Slippery elm | tau = 0.27; *** | 0.39; ** | YES, 12 | January (5.78) | December (-4.28) | 10.05 |
| Spirulina | tau = 0.61; *** | 2.54; *** | YES, 12 | March (4.49) | December (-10.37) | 14.86 |
| ß-Alanine | tau = 0.73; *** | 4.05; *** | YES, 12 | January (5.83) | December (-6.45) | 12.28 |
| Stevia | tau = 0.52; *** | 2.90; *** | YES, 12 | May (4.85) | December (-13.73) | 18.59 |
| Sucrase | tau = -0.37; *** | -1.53; *** | YES, 12 | October (13.79) | August (-16.60) | 30.39 |
| Synbiotics | tau = 0.12; 0.024 | 0.92; *** | YES, 12 | February (5.16) | July (-3.32) | 8.47 |
| Taurine | tau = 0.13; 0.02 | 0.16; 0.157 | YES, 12 | April (4.66) | December (-10.44) | 15.09 |
| Theanine | tau = 0.82; *** | 3.55; *** | YES, 12 | September (2.34) | December (-6.48) | 8.82 |
| Thiamine | tau = 0.85; *** | 2.44; *** | YES, 12 | April (3.77) | December (-8.17) | 11.94 |
| Threonine | tau = -0.29; *** | -1.40; *** | YES, 12 | October (7.90) | December (-9.06) | 16.96 |
| Tryptophan | tau = -0.33; *** | -0.58; *** | YES, 12 | November (29.91) | July (-5.80) | 35.71 |
| Turmeric | tau = 0.89; *** | 5.12; *** | YES, 12 | January (4.68) | June (-2.20) | 6.89 |
| Tyrosine | tau = -0.57; *** | -1.32; *** | YES, 12 | May (5.71) | December (-8.33) | 14.04 |
| Valerian | tau = 0.64; *** | 0.88; *** | YES, 12 | July (2.92) | December (-2.67) | 5.60 |
| Valine | tau = -0.17; ** | -0.26; 0.065 | YES, 12 | October (7.62) | July (-10.17) | 17.79 |
| Vanadium | tau = -0.64; *** | -1.92; *** | YES, 12 | October (2.24) | December (-2.78) | 5.02 |
| Velvet bean | tau = 0.65; *** | 2.43; *** | YES, 12 | March (2.66) | December (-4.44) | 7.09 |
| Vitamin A | tau = -0.17; ** | -0.45; *** | YES, 12 | November (8.63) | July (-11.64) | 20.26 |
| Vitamin B-6 | tau = 0.9; *** | 3.01; *** | YES, 12 | March (3.88) | December (-7.24) | 11.13 |
| Vitamin C | tau = 0.68; *** | 2.43; *** | YES, 12 | October (4.92) | July (-7.57) | 12.49 |
| Vitamin D | tau = 0.97; *** | 4.86; *** | YES, 12 | February (6.68) | July (-6.34) | 13.01 |
| Vitamin E | tau = 0.45; *** | 0.49; *** | YES, 12 | November (4.93) | July (-3.05) | 7.98 |
| Vitamin K | tau = 0.68; *** | 1.37; *** | YES, 12 | April (5.22) | December (-9.10) | 14.33 |
| Vitamin K2 | tau = 0.92; *** | 6.11; *** | YES, 12 | February (4.00) | August (-2.52) | 6.51 |
| Whey protein | tau = 0.76; *** | 4.44; *** | YES, 12 | January (4.82) | December (-13.81) | 18.63 |

| | | | | | | |
|---|---|---|---|---|---|---|
| Yohimbe | tau = -0.85; *** | -3.43; *** | NO, - | - | - | - |
| Zeaxanthin | tau = 0.2; *** | 0.42; 0.024 | YES, 12 | September (3.74) | December (-7.08) | 10.82 |
| Zinc | tau = 0.67; *** | 2.39; *** | YES, 12 | February (3.17) | December (-4.38) | 7.56 |

Dataset
